# Probenecid arrests the progression of pronounced clinical symptoms in a mouse model of multiple sclerosis

**DOI:** 10.1038/s41598-017-17517-5

**Published:** 2017-12-08

**Authors:** Nadine Hainz, Sandra Wolf, Artjom Beck, Stefan Wagenpfeil, Thomas Tschernig, Carola Meier

**Affiliations:** 1 Dept. of Anatomy and Cell Biology, Saarland University, Homburg/Saar, Germany; 2 Institute for Medical Biometry, Epidemiology & Medical Informatics, Saarland University, Homburg/Saar, Germany

## Abstract

While it has been established that Probenecid (PBN) prevents the onset of experimental autoimmune encephalomyelitis (EAE) in mice, it is not clear whether it has any effect on already manifest EAE. The aim of this study was therefore to analyze the therapeutic effect of PBN in pronounced EAE. Mice with manifest clinical symptoms of EAE were either treated with PBN or solvent for 20 days, or they were left untreated. The clinical symptoms were monitored daily. Inflammation, demyelination and oligodendrocyte numbers were determined in the spinal cord. We were able to demonstrate that PBN not only significantly prolonged survival but also prevented the progression of clinical symptoms in the EAE model of multiple sclerosis. In addition, we were able to show that PBN reduced inflammation, T cell infiltration and oligodendrocyte cell loss. PBN was previously shown to inhibit – among other targets – pannexin channels. As pannexin channels provide conduits for ATP, are associated with the inflammasome, and act as “find me-signals” in the process of apoptosis, inhibition of pannexins via PBN might contribute to the PBN-effects observed in this study. The beneficial and therapeutic effects of PBN in the context of EAE demonstrate an intriguing link between PBN and neuroinflammation, which might foster translational interest.

## Introduction

Characteristic pathologic features of MS are inflammation and demyelination resulting, along with other symptoms, in the loss of motor function. Experimental autoimmune encephalomyelitis (EAE) is a commonly used murine model of multiple sclerosis (MS), which mimics most of the features found in this disease^1^. T cells and activated microglia are known to participate in inflammatory MS processes. Neurodegenerative changes include demyelination, followed by the loss of oligodendrocytes.

The pannexin (Panx)-1 channel is expressed in both neural and immune cells, the latter including macrophages and T cells^2,3^. Panx1 is an ATP channel^4^, and as such also involved in apoptosis^5^. Extracellular ATP released by Panx1 channels was shown to modulate both the initiation and the clearance of inflammatory responses^6^. Intracellularly, Panx1 channels are linked to the cytoplasmic inflammasome^2^. Probenecid (PBN) is a clinically-approved drug, which – among other targets – inhibits the Panx1 channel and prevents activation of the inflammasome^2,7^. Very recently, it was shown that the microglia Panx1-mediated ATP release is one major contributor to opiate withdrawal symptoms and that, in turn, inhibition of Panx1 via PBN reduced withdrawal symptoms in rodents^8^. In EAE, PBN prevented the onset of clinical symptoms in mice^9^. However, it is not clear whether PBN has any effect on already manifest EAE. The aim of this study was therefore to analyze a potential therapeutic effect of PBN in pronounced EAE. Here, we show that EAE does not progress in PBN-treated animals and that these effects are associated with reduced inflammation and increased numbers of oligodendrocytes.

## Results

### PBN-treatment arrests clinical symptoms and prolongs survival in mice with manifest EAE

EAE was induced in all mice and clinical symptoms were monitored daily from the start of immunization (day 0). After an initial period basically free of clinical symptoms, clinical scores began to increase in all animals from day 9 onwards. Animals of all three experimental groups displayed a similar development of clinical symptoms up to day 15, when the mean score of all animals reached 2.23 ± 0.06.

**Figure 1:**
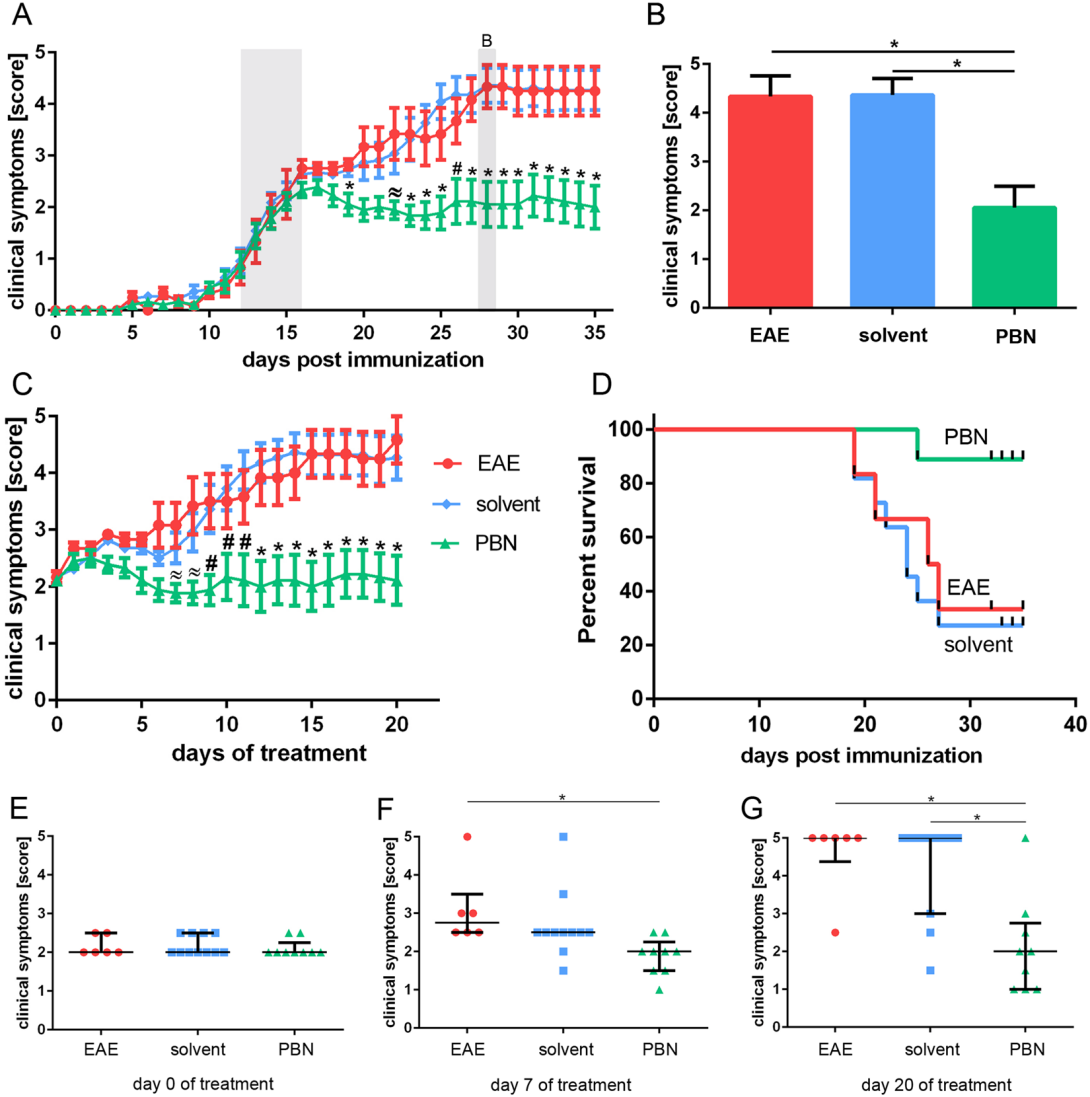
PBN treatment curbs EAE symptoms. (**A**) Clinical score monitored during EAE (induction at day 0). Treatment with PBN [green; n = 9] or solvent [blue; n = 11] commenced when the individual score reached 2 (between days 12 and 16 after induction, gray zone). Animals of the EAE group [red; n = 6] were left untreated. Shown are means + /− standard error of the mean. For analysis of PBN effects on individual experimental days, ANOVA analysis followed by Tukey’s post-hoc test was performed. p < 0.05 was considered significantly different, illustrated by ≈ PBN versus EAE, # PBN versus solvent, and * PBN versus EAE and solvent. (**B**) Illustration of scores on day 28 (gray bar in A), showing the mean plus standard error of the mean. *p < 0.05. (**C**) Clinical symptoms of PBN- [green; n = 9] and solvent- [blue; n = 11] treated animals as well as untreated EAE animals [red; n = 6] (days of treatment). Scores were monitored after the individual onset of treatment (defined as day 0 of treatment). Shown are means + /− standard error of the mean. p < 0.05 was considered significantly different, illustrated by ≈ PBN versus EAE, # PBN versus solvent, and * PBN versus EAE and solvent. (**D**) Survival of EAE [red] and solvent [blue] animals was significantly lower than that of PBN [green] animals. (**D–G**) For illustration of the development of clinical symptoms in the experimental groups EAE, solvent, and PBN, the median of scores [1. quartile; 3. quartile] is demonstrated at the starting point of treatment, i.e. day 0 (**E**), at day 7 of treatment (**F**) and at the final point of the experiment, i.e. day 20 (**G**). *p < 0.05.

To analyze the therapeutic effect of PBN in manifest EAE, PBN application was started when mice reached score 2. At a clinical score of 2, EAE symptoms were severe in that mice displayed one-sided hindlimb paralyses at minimum. In an initial approach, PBN was administered at a concentration of 100 mg/kg body weight for the following 10 days, i.e. at the dosage previously successfully used to prevent the onset of EAE^9^. At this concentration and experimental duration, however, neither survival nor clinical scores differed significantly between treated and untreated animals (data not shown). Thus, in the succeeding experiment, 250 mg/kg body weight PBN was applied by intraperitoneal injection once daily (PBN group) for the next twenty days. In the two control groups, animals received no treatment at all (EAE group) or injections of the solvent only (solvent group). After the start of treatment, the symptoms in mice of the solvent and EAE groups proceeded to develop similarly up to the end of the experiment, reaching a mean score of 4.26 ± 0.01 in both groups. General estimated equation (GEE) analysis confirmed that differences between these two groups, EAE and solvent, were not significant (p = 0.836). In contrast, the scores in the PBN group stabilized on day 16 and remained unchanged for the rest of the experiment, indicating that PBN treatment has an inhibitive influence on symptom development (Fig. 1A).

In general, the scores of PBN animals differed significantly from those of the EAE and the solvent groups as evidenced by linear regression (GEE) analysis over the complete course of the experiment (p < 0.001). Detailed analysis of individual experimental phases (phase I: days 0 to 12; phase II: days 13 to 17; phase III: days 18 to 35) revealed a significant reduction of mean clinical scores in animals of the PBN group as compared to those of the solvent and EAE groups during the third phase (p < 0.01). Scores at day 28 (mean +/− SEM) are illustrated exemplarily in Fig. 1B, illustrating significantly lower clinical scores in PBN-treated animals than in animals from the EAE and solvent groups.

As the asynchronous onset of clinical symptoms in immunized mice resulted in individual treatment starting points (Fig. 1A), scores were also analyzed in relation to the duration of treatment (Fig. 1C,E–G). Treatment commenced at an individual minimal score of 2 (Fig. 1E) and was continued for 20 days. The clinical symptoms in PBN-treated mice were astonishingly stable throughout the whole treatment period. Inspection of individual treatment days (median; 1. quartile; 3. quartile) illustrates the distribution and the changes within experimental groups even more clearly (Fig. 1E–G). In contrast to PBN animals, clinical symptoms continuously worsened in all animals of the EAE and solvent groups, resulting in significantly higher scores (both groups compared to PBN) from day 12 of treatment onwards (Fig. 1C, E–G).

Animals in the PBN group survived significantly longer than those in the solvent or EAE groups (Fig. 1D). Till the end of the experiment (day 35 post immunization), only one mouse out of the PBN group had died (on day 25), whereas less than 65% of solvent mice and only 50% of EAE mice were alive on days 25 and 26, respectively (Fig. 1D).

### PBN does not affect body weight or activity of control animals

**Figure 2:**
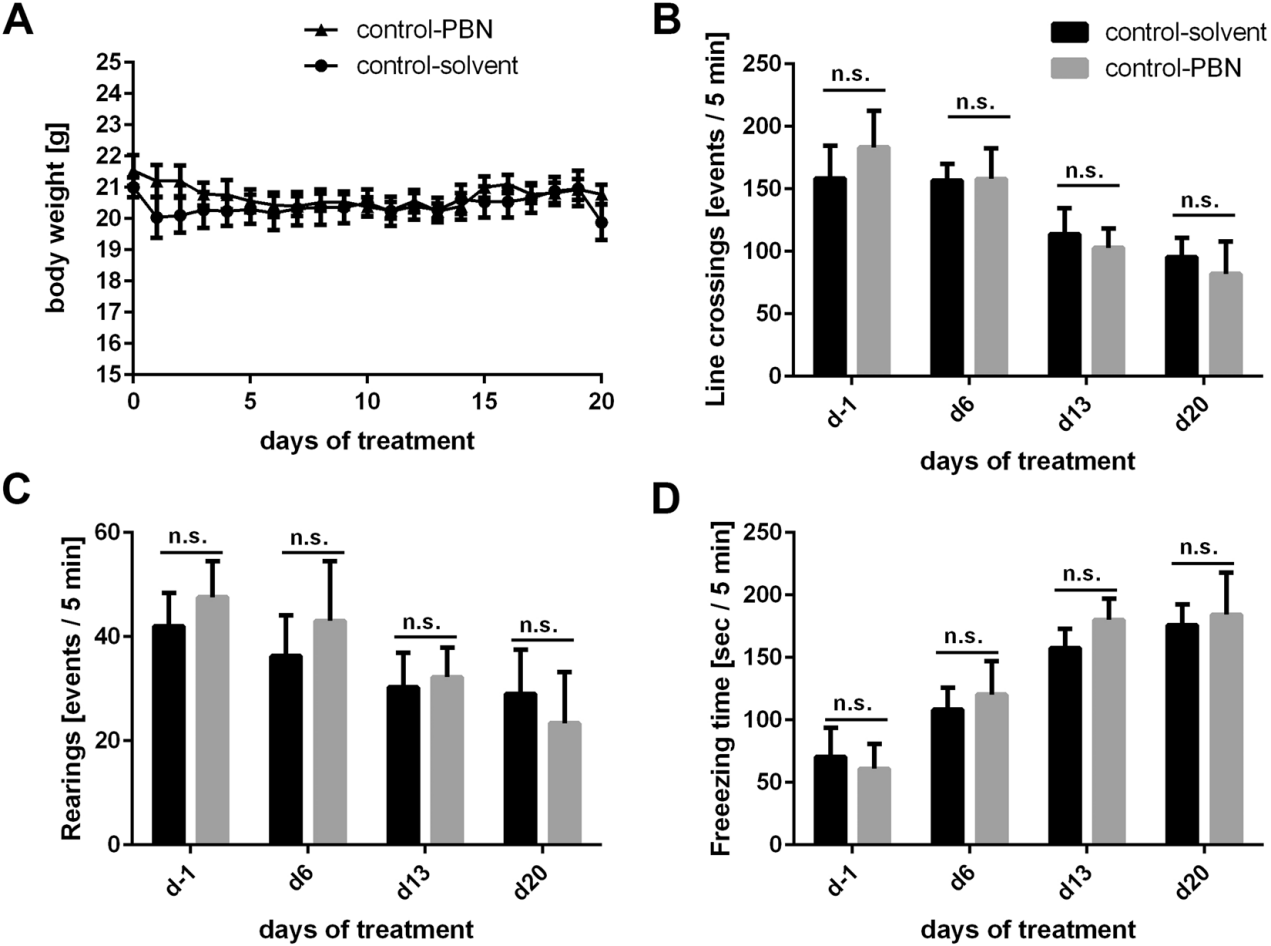
PBN does not affect weight or activity of control mice. (**A**) C57BL/6 mice were injected daily with either solvent [circles; n = 4; control-solvent] or PBN (250 mg/kg BW) [triangles; n = 5; control-PBN] for 20 days. The weight was monitored daily and did not show significant differences between the two groups as determined by GEE analysis (p ≥ 0.05). (**B**–**D**) An open field test was employed to assess activity of solvent- (control-solvent; black columns) and PBN-injected (control-PBN; gray columns) animals. Monitoring time was 5 min. Open field analysis was performed one day prior to the first injection (d–1), and once weekly after that (d6, d13, d20). Shown are means + /− standard error of the mean. (**B**) The number of line crossings did not differ between animals of both groups (p ≥ 0.05). (**C**) The number of rearings was similar between animals of both groups (p ≥ 0.05). (**D**) The freezing time did not differ between both treatment groups during the 5 min observation interval (p ≥ 0.05). n.s. not significant.

To exclude potential side effects of PBN at the dosage applied (250 mg/kg body weight), we injected control mice the same way as those of the EAE experiment. Mice were injected intraperitoneally with either PBN (n = 5) or solvent (n = 4) for 20 days. General behavior of mice and their weight were monitored daily; the activity pattern was analyzed by open field analysis once weekly. The body weight was similar in all animals and stayed stable over the duration of the experiment irrespective of treatment (Fig. 2A). Open field analysis did not reveal any significant difference between animals of both experimental groups with respect to activity: All parameters investigated, i.e. the time animals moved (indicated by the number of line crossings; Fig. 2B), the number of rearings (Fig. 2C) or the freezing time (Fig. 2D) did not differ between both experimental groups, indicating that PBN did not affect any of these parameters in control animals.

### PBN-treatment reduces inflammation and increases oligodendrocyte numbers in EAE animals

**Figure 3:**
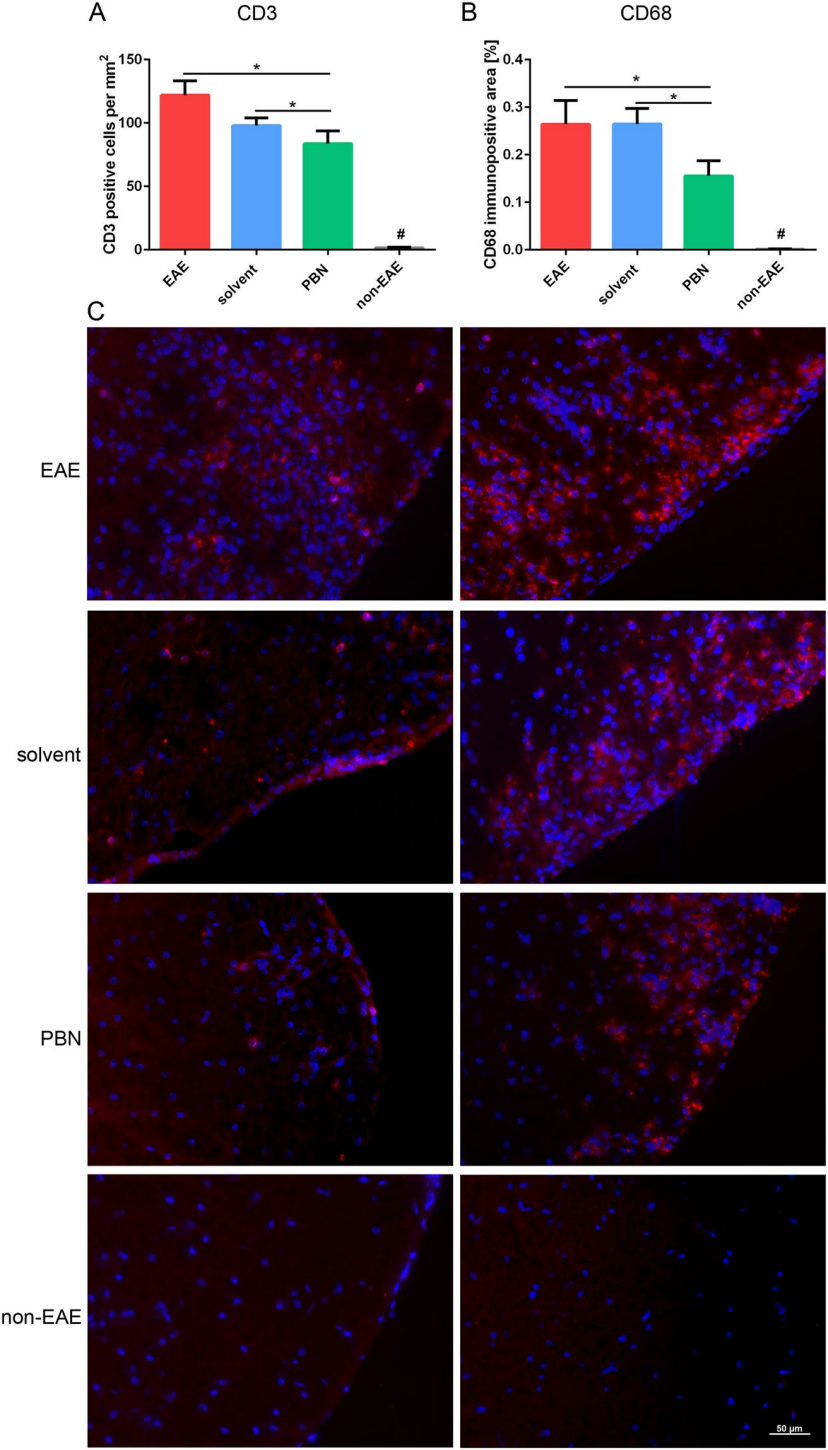
(**A**–**C**) Immunofluorescence analyses labeling CD3 and CD68 were performed on spinal cord sections upon completion of the experiment. (**A**) The number of T cells (CD3-immunopositive) was significantly lower in PBN animals than in mice of solvent and EAE groups. * p < 0.05 as indicated. In spinal cords of non-EAE control animals, CD3-immunopositive cells were mostly absent and numbers were significantly lower than in animals of the EAE, solvent and PBN groups (# p < 0.05 compared to all other experimental groups). (**B**) Microglia activation (CD68-immunopositive) was significantly lower in PBN animals than in mice of solvent and EAE groups. * p < 0.05 as indicated. Non-EAE control animals did not show any CD68-immunopositive area in spinal cords (# p < 0.05 compared to all other experimental groups). (**C**) Exemplary photographs of CD3 (left panel; red) and CD68 immunofluorescence (right panel; red) of the anterior spinal cord from all experimental groups as indicated. Nuclei were stained by DAPI (blue).

In view of the observation that the motor function in animals of the EAE-PBN group was better than in the other groups, we analyzed morphologic changes in the ventral corticospinal tract. Lymphocyte infiltration, assessed by the immunofluorescence labeling of T cells (CD3; Fig. 3A,C), and inflammation, determined by the detection of activated microglia (CD68; Fig. 3B,C), were significantly reduced in animals of the PBN group as compared to those of the solvent and EAE groups. The reduced number of cellular infiltrates is also reflected by a reduction of DAPI-stained nuclei in PBN-treated animals. The number of CD3-positive cells as well as the CD68-immunopositive area correlated positively with the severity of EAE, i.e. the score (Spearman-Rho correlation coefficient of CD68 and score 0.458 and of T cells and score 0.446).

**Figure 4:**
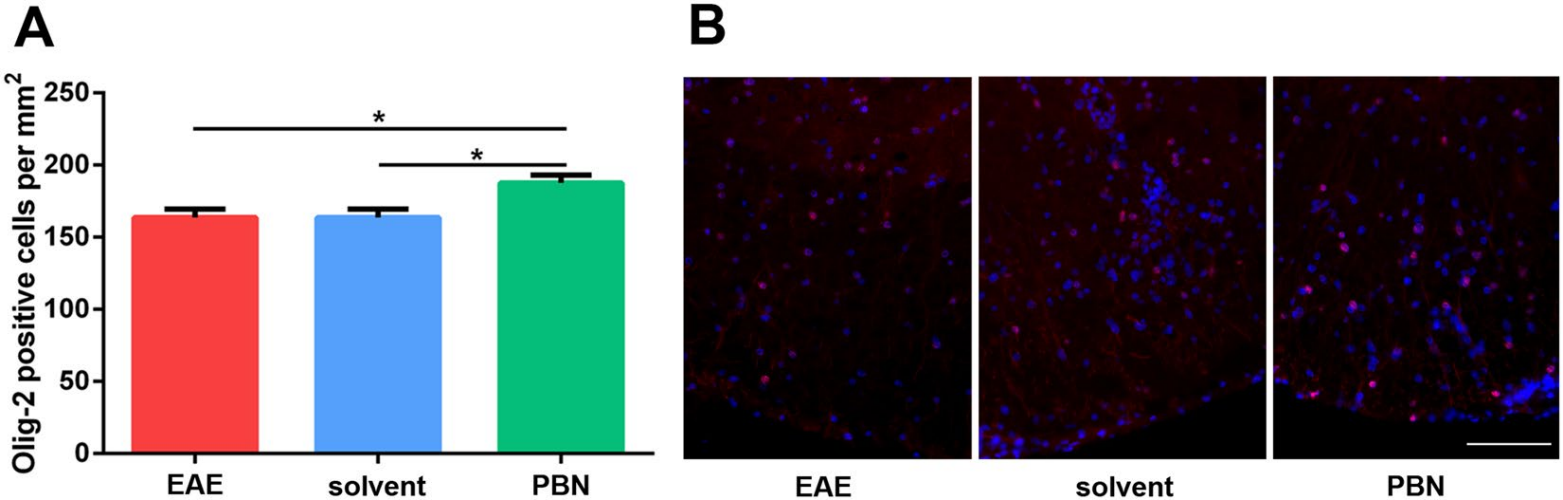
(**A**) Immunofluorescence analyses labeling the oligodendrocyte marker protein Olig2 were performed on spinal cord sections upon completion of the experiment. Oligodendrocyte cell number was higher in PBN-treated mice as compared to both other groups. * p < 0.05. (**B**) Illustration of Olig-2 immunofluorescence (red). Nuclei were stained by DAPI (blue). Scale bar 100 µm.

**Figure 5:**
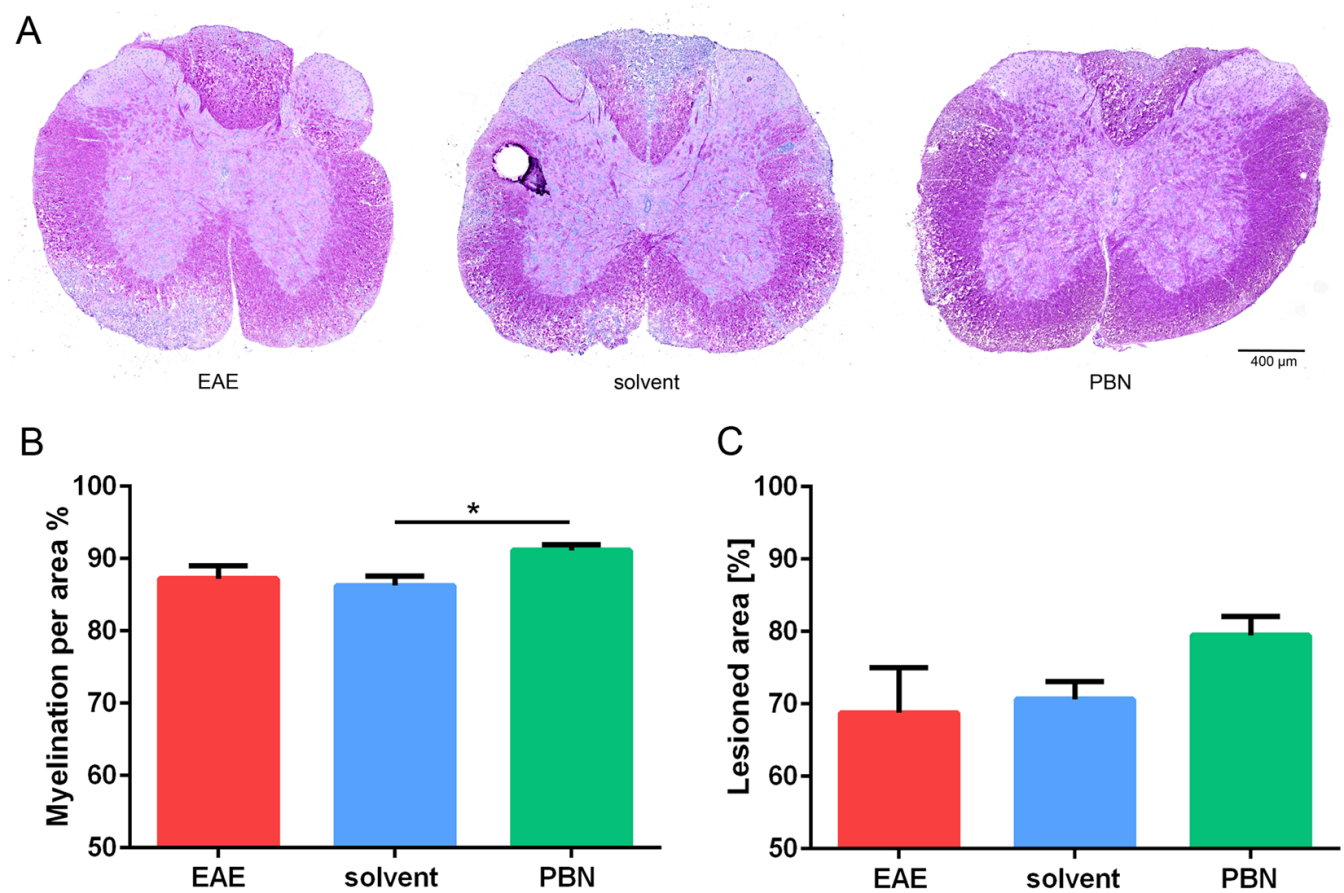
(**A**) PAS histological staining of spinal cord cross sections, illustrating the degree of myelination/demyelination in spinal cord white matter. Spinal cords were derived from non-treated EAE animals (EAE), solvent-injected animals (solvent) and PBN-treated animals (i.p. application of 250 mg/kg body weight; PBN). Myelin is stained purple; white matter areas devoid of myelin appear blue-gray. (**B**) Quantification of PAS staining reveals a mild increase of the PAS-stained myelinated area in ventrolateral tracts of the spinal cord in the PBN group. Illustrated are means + /− SEM; values of p < 0.05 were considered statistically significant. *p < 0.05 (**C**) The lesioned area was quantified in HE-stained sections.

Olig-2 immunofluorescence was performed to determine oligodendrocyte numbers and thus to analyze oligodendrocyte cell loss (Fig. 4). Olig-2 immunolabeling revealed significantly fewer oligodendrocytes in animals of solvent and EAE groups compared to those of the PBN-treated group. The increase in oligodendrocyte numbers upon PBN treatment was also reflected in the appearance of PAS-stained spinal cord sections (Fig. 5A,B), whereas differences in the size of the lesion area in HE-stained spinal cord were not significant (Fig. 5C).

## Discussion

It had previously been shown that PBN prevents the onset of EAE^9^. Here, we analyzed the effects of PBN in manifest EAE. This is not only an approach, which is novel in the analysis of the effects of PBN *per se*, but is also a strategy, which differs distinctively from other pharmacological studies conducted in the EAE model: Whereas the majority of studies are carried out prior to the onset of symptoms, in our present approach PBN-treatment is installed when disease symptoms are definitively established. Thus, application of PBN commenced when animals had developed score 2, characterized by pronounced hindlimb paralysis. At the pathophysiological level, this score correlates with T cell infiltration, microglia activation and demyelination. In consequence, the inhibition of Panx1 channels via PBN kicks in at a time-point, when downstream mechanisms like inflammasome activation have already been triggered. The results of this study imply that PBN is still able to put pathophysiological events to a hold, evidenced by the observed stabilization of the clinical score. In this context, it is important to point out that PBN at the concentration applied did not affect weight or activity of control mice. Together with the data obtained from clinical application of PBN in humans, this data render adverse effects of PBN unlikely.

One major contributor to disease progression is the activation of microglia. In this study, the number of activated microglia cells, determined by CD68 immunofluorescence, was significantly reduced in PBN-treated EAE animals as compared to non-treated or solvent-injected EAE animals. This observation is in line with studies on the preventive application of PBN in EAE mice^9^ and on PBN treatment in brain ischemia and reperfusion^10^. One important factor mediating the chemotactic action on microglia cells and macrophages is the insult-related release of nucleotides, particularly that of adenosine triphosphate (ATP)^5,11^, via Panx1 channels^5^. Data on the pharmacologic inhibition of Panx1 channels, for instance by carbenoxolone or probenecid, complement this finding in that Panx1 inhibition was shown to result in reduced monocyte recruitment by apoptotic cells^5^.

Besides chemotactic action on microglia cells, extracellular ATP also causes lymphocyte migration through blood vessel walls and therefore promotes lymphocyte entry into the CNS^12^. In the context of an autoimmune disorder like multiple sclerosis, this process – and the inhibition thereof – might be crucial for the progression of the disease. Velasquez *et al*. already demonstrated fewer infiltrating CD4-positive T cells in spinal cords of EAE mice with genetic deletion of Panx1 as compared to EAE wild-type mice^13^. In our study, the number of CD3-positive T cells was significantly reduced in PBN-treated EAE animals. Schenk *et al*. were able to demonstrate an influence of Panx1 on the subsequent step of T cell immunology, i.e. their activation^14^. Interestingly, the authors refer to an autocrine mechanism of T cell activation via the Panx1-mediated ATP release of T cells themselves.

In addition to paracrine and autocrine effects downstream of Panx1, the intracellular connection of Panx1 to the inflammasome is also of prime importance in the onset and the perpetuation of inflammatory conditions. Studies focusing on the role of the inflammasome in EAE showed that its inhibition protected from or delayed disease onset^3,15^. Thus, the inflammasome inhibitor PBN is a prime candidate for dual action directed at neural and inflammatory cells^2,7^. One example for a direct effect of PBN on neural cells are astrocytes, which showed better viability and reduced inflammasome activity under hypoxic conditions^16^. The observation of this study that the number of oligodendrocytes was increased in the spinal cords of mice treated with PBN demonstrates a direct – or indirect – impact on neural cells.

It will be of major importance to dissect the effects of PBN and to elucidate its molecular targets in the context of EAE. Although PBN clearly inhibits Panx1 channel activity^7^, its direct inhibitory effects on the purinergic P2X7 receptor^17^ and the inhibition of organic anion transporters^18^ also have to be taken into account. Interestingly, application of various pharmacologic inhibitors in EAE consolidate the importance of the Panx1 – P2X7 axis in the pathology of demyelinating conditions: Inhibition of P2X7^19^, pharmacologic inhibition via carbenoxolone or mefloquine – both of which inhibit connexin and pannexin channels^3,20,21^ –, as well as Panx1 deficiency in mice^3^, all prevent or delay the onset of EAE.

We previously demonstrated that PBN is able to cross the blood-brain-barrier^22^ and can therefore act locally at the side of inflammatory lesions in the CNS. Thus, PBN treatment might complement other pharmacologic strategies, which primarily aim at preventing lymphocyte exit from lymphoid organs or lymphocyte entry into the CNS^23–25^. To sum it up, a dual action of PBN on immune cells as well as on oligodendrocytes has been demonstrated in this study. Both effects might possibly be interlinked in that a reduction of activated microglia will cause a reduction in inflammatory cytokines and therefore in immigrating lymphocytes – which in summary might provide a regenerative environment for oligodendrocytes. The mild reduction of demyelination following probenecid treatment has recently also been described in cuprizone-induced demyelination^26^. In summary, these data substantiate the great importance of PBN for future therapeutic strategies – particularly in view of the fact that it has already been approved for clinical use.

## Material and Methods

### Experimental autoimmune encephalomyelitis (EAE)

To induce EAE, 12-week-old C57BL/6 mice (Charles River, Sulzfeld; Germany) were immunized using the Hooke Kit EK-2110 (Hooke Laboratories; MA; USA) according to the manufacturer’s instructions. Briefly, 200 µl MOG emulsion (containing the MOG peptide of amino acids 35 to 55 of the myelin oligodendrocyte glycoprotein, MOG35-55 and complete Freund’s adjuvant) 10 mg/ml) was injected subcutaneously into the upper and lower back. Four hours after immunization and 24 hours later, 200 µl of a pertussis toxin solution (250 ng in 200 µl NaCl) was injected for enhancement of the immune response. Clinical EAE symptoms were monitored daily and assessed in neurological scores. Criteria for the assignment to certain scores were score 0 = no symptoms; score 0.5 = partial tail paralysis; score 1 = limp tail; score 2 = partial hind limb paralysis; score 3 = complete hind limb paresis; score 4 = fore- and hind limb paresis; score 5 = death.

When score 2 was reached, characterized mainly by one-sided hindlimb paralysis, the mice were randomized into three groups. The first group (EAE; n = 6) was left untreated, the second group (PBN; n = 9) received PBN (250 mg/kg body weight, intraperitoneal injection) and the third group (solvent; n = 11) received solvent only.

The PBN injection solution (pH 7.3) was prepared at a total volume of 15 ml, containing 375 mg PBN (w/v), 1500 µl 1 N NaOH (v/v), 1500 µl 1 M Tris, 12 ml 0.9% NaCl in dist. H_2_O, and, for pH adjustment, 2 N HCl. Accordingly, solvent treatment was performed via injection of the solvent only, always with the same pH.

The mice were sacrificed after 20 days of therapy or upon pre-defined no-go criteria being reached. If a mouse died during the experiment or was taken out of the experiment because no-go criteria occurred, its score was rated 5 (=death) for the rest of the experiment. Mice that had been treated for 20 days were classified under their final score for the rest of the experiment. Animals were sacrificed at the end of the experiment and spinal cords were dissected. All animal experiments were in compliance with the German Animal Protection Law and the German Guide for the Care and Use of Laboratory Animals and were approved by the governmental Animal Care Committee of the Saarland, Germany.

### PBN control experiments

To determine whether PBN affected the weight or activity of animals, control mice received intraperitoneal injections of either PBN solution (control-PBN; n = 5) or solvent (control-solvent; n = 4) for 20 days. Sex and age of control animals, the concentration of PBN (250 mg/kg body weight) and the number of daily injections (=20) were identical to those employed during EAE-treatment. The PBN solution and solvent solution were prepared as described above. During the experiment, the body weight and general behavior of mice in their cages was monitored daily. Animal experiments were in compliance with the German Animal Protection Law and the German Guide for the Care and Use of Laboratory Animals and were approved by the governmental Animal Care Committee of the Saarland, Germany.

### Open field analysis

Open field analysis was performed in a box measuring 38 × 38 cm, which was equipped with rectangular lines on the floor. Animals were placed in the box and their behavior was analyzed for 5 min. Behavior of all mice was determined one day before injections were started (d-1). Mice were then randomized to two experimental groups (control-PBN; n = 5 and control-solvent; n = 4) and the open field test was repeated three times (once a week) during the experimental duration (d7, d13, d20). Individual mice were placed into the center of the open field box and were filmed and observed for 5 minutes. Afterwards, video sequences were analyzed by a blinded investigator. Parameters like the number of line crossings and number of rearings as well as the duration of freezing periods were quantified.

### Immunohistochemistry

Parts of the spinal cords were cryoprotected in Tissue Tek® compound (Weckert Labortechnik, Kitzingen) in the gaseous phase of liquid nitrogen. Cryosections of 8 µm thickness were prepared (cross sections), air dried for 30 min and then fixed in −20 °C cold 100% acetone for 10 min. After inhibition of non-specific binding sites (incubation in 10% normal goat serum, 0.1% TritonX100 in PBS for two hours), incubation with primary antibodies, diluted in 10% normal goat serum and 0.1% TritonX100 in PBS, was performed at 4 °C for 16 hours. Four cross-sections from the spinal cord of each mouse (EAE n = 5, PBN n = 8, solvent n = 9, non-EAE n = 3) were immunolabelled with antibodies against CD3 (1:100; rabbit polyclonal antibody A0452; Agilent Technologies, Hamburg, Germany) and CD68 (1:100; rat monoclonal antibody ab53444; Abcam, Cambridge, UK). Spinal cords of age- and sex-matched untreated, non-immunized mice (non-EAE) served as experimental controls. Omission of primary antibodies served as negative controls. Antibodies were applied according to the manufacturer’s instructions; specificity of antibodies was previously demonstrated^27^. Immunolabelling of oligodendrocytes was performed using Olig-2 antibodies (DF308, Dana-Farber Cancer Institute, Boston, USA), developed and characterized by Ligon *et al*., and applied at a dilution of 1:20,000^28^. The sections were then washed three times with PBS for 10 min each time, followed by blocking in 0.2% bovine serum albumin (BSA) in PBS for 30 min. After that, the sections were incubated with the appropriate Alexa Fluor®- conjugated secondary antibody (goat-anti-rabbit Alexa Fluor® 568; goat-anti-rat Alexa Fluor® 568; Thermo Fisher Scientific, 1:3,000) at room temperature for one hour. Sections were embedded using DAPI-Fluoromount G® embedding medium (Biozol, Eching, Germany). Photographs of the antero-lateral tract of spinal cord sections were taken with identical exposure times using the fluorescence microscope Observer Z.1 (Zeiss, Göttingen, Germany) with Axio Vision 4.8 software (Zeiss). CD68 immunofluorescence was quantified on three sections per animal using the ImageJ software (National Institutes of Health (NIH), USA). After conversion of photographs into 8 bit images, an identical threshold was set and used for analysis of all pictures. With this threshold, the fluorescent points were represented in black, whereas non-labeled points were shown in white. Quantification of these data was performed in a blinded fashion by calculating the percentage of the labeled area (black) in relation to the whole area of the picture. For quantification of CD3 and Olig2 immunosignals, the number of immunopositive cells per area was determined in photographs of three spinal cord sections per animal (EAE n = 5, PBN n = 8, solvent n = 9, non-EAE n = 3). Quantification of immunofluorescence was performed in a blinded fashion.

For manuscript figures, photographs were assembled using Adobe Photoshop (CS4 extended version); brightness and contrast were adjusted in assembled photographs.

### Histology

For histological staining of myelin, periodic acid staining (PAS staining) was performed on cryosections of 8 µm thickness. After incubation in periodic acid (1% in dist. H_2_O; 5 min), sections were briefly rinsed in dist. H_2_O and subsequently incubated in Schiff reagent in the dark for one hour. Incubation in sulfurous acid (3 times 5 min) completes the reaction. Afterwards, hematoxylin staining of nuclei was performed according to Ehrlich (15 min incubation). After several rinses in tap water, the sections were dehydrated by increasing alcohol concentrations 85 min each), subsequently incubated in xylol (3 times 5 min) and mounted in Roti Histokitt mounting medium (Roth, Karlsruhe, Germany). Quantification was performed on two sections per animal using the ImageJ software (NIH, USA). After conversion of photographs into 8 bit images, an identical threshold was set and used for analysis of all pictures. With this threshold, the PAS-positive areas were represented in black, whereas non-stained areas were shown in white. Quantification of these data was performed in a blinded fashion by calculating the percentage of the labeled area (black) in relation to the whole area of the picture.

Hematoxylin-Eosin staining was performed on cryosections of 8 µm thickness. Sections were fixed in 100% ethanol (−20 °C) for 5 min, followed by a 2 min rinse in dist. H_2_O and subsequent incubation in hematoxylin solution according to Ehrlich for 5 min. Sections were then rinsed for 10 min in tap water (differentiation of blue color), rinsed in dist. H_2_O and incubated in 0.1% Eosin in dist. H_2_O for 10 sec. Following incubation in 80% 2-propanol for 3 min (differentiation of red color), sections were incubated in increasing alcohol concentrations, followed by 3 times 5 min incubation in Xylol, and mounted in Roti-Histokitt mounting medium. Quantification of the lesion area was performed on two sections per animal using the ImageJ software (NIH, USA).

### Statistical analyses

Generalized Estimation Equation (GEE) analysis was performed using SPSS Statistics 19 (IBM). All other statistical analyses were performed using GraphPad Prism 6 Software (GraphPad, La Jolla, USA). Two-sided p values < 0.05 were considered statistically significant. The Log-rank (Mantel-Cox) test was used for survival analysis. One-way ANOVA followed by Tukey’s multiple comparisons test was used to analyze the scores on individual days; GEE analysis was performed for comprehensive comparison of treatment groups. Immunohistochemical data were statistically analyzed using Kruskal-Wallis followed by Dunn’s multiple comparisons test. Correlations were determined employing Spearman-Rho analysis using SPSS Statistics 19 (IBM).
